# Multichamber magnetic capsule robot for selective liquid sampling and drug delivery

**DOI:** 10.1093/nsr/nwaf400

**Published:** 2025-09-19

**Authors:** Zehao Wu, Xianli Wang, Yang Lu, Qingsong Xu

**Affiliations:** Department of Electromechanical Engineering, Faculty of Science and Technology, University of Macau, Macau, China; Department of Electromechanical Engineering, Faculty of Science and Technology, University of Macau, Macau, China; Department of Mechanical Engineering, The University of Hong Kong, Hong Kong, China; Department of Electromechanical Engineering, Faculty of Science and Technology, University of Macau, Macau, China

**Keywords:** magnetic capsule robot, multichamber, integrated biopsy and medication, liquid sampling, drug delivery

## Abstract

Ingestible capsule robots provide a minimally invasive approach to diagnosing and treating gastrointestinal diseases. However, current capsule robots cannot realize both diagnosis and treatment because of the conflict between manufacturing simplicity and functional enhancement. Here, we present a multichamber magnetic capsule robot (macabot) to achieve selective liquid sampling and drug delivery via one-time oral intake. The engineered multichamber macabot realizes selective opening of four individual chambers in specified directions triggered by a gradient magnetic field. Such functions are decoupled from its rolling locomotion driven by a rotating magnetic field. The multichamber macabot can be split into single-chamber modules to navigate narrow intestine tracts with higher pH values. The experimental results demonstrate the capabilities of the macabot for targeted delivery, sealing, active navigation, liquid sampling and release operations. Successful validation was achieved for both *in situ* generation of a shape-adaptive hydrogel patch for targeted local deployment, and *ex vivo* liquid sampling and release under ultrasound imaging guidance.

## Teaser

A multichamber magnetic capsule robot enables selective liquid sampling and drug delivery in gastrointestinal tract via one-time oral intake.

## INTRODUCTION

With the advancement of modern society, changes in dietary structures and increased work-related stress have caused a gradual rise in the incidence of gastrointestinal (GI) diseases worldwide [1,2]. Common types of GI diseases include gastritis, gastric ulcers, inflammatory bowel disease (IBD) and digestive tract cancers. The incidence and number of cancer-related deaths from GI diseases account for more than one-quarter and one-third of all cancer cases, respectively [3]. Currently, the treatment methods for GI diseases mainly include drug therapy and surgical therapy. However, these methods exhibit several shortcomings, such as poor curative effects, noticeable side effects and insufficient individualization. Researchers are developing more efficient diagnostic and therapeutic medical tools for potential use in the GI tract [4–7]. A capsule device is a typical mechanism for executing drug delivery [8], disease diagnosis [9] and biopsy [10] tasks. This approach offers the advantage of high compliance [11,12] and has become the most prevalent GI medical tool. Traditional capsule devices execute medical tasks depending on intestinal peristalsis and environmental conditions, which are passive and uncontrollable mechanisms characterized by instability and imprecision. In drug delivery, passive mechanisms may cause drugs to leave target sites. Thus, to maintain therapeutic efficacy, it is necessary to administer a greater quantity of the drug than needed, thereby increasing the adverse side effects [13]. Furthermore, this instability induces blind spots during endoscopic procedures [14], increases the risk of retention within the body [15] and restricts the ability to conduct biopsies. Therefore, it is desirable to introduce active capsule devices to realize targeted movement and controlled execution for localized treatment [16–20].

Magnetic fields offer several advantages regarding active capsule device actuation, including deep penetration, safety and controllability [21–26]. Magnetic field actuation also reduces the requirements of complex mechanisms and power supplies, thereby decreasing the device’s overall size and complexity. In the literature, several magnetic capsule robots have been reported [27,28] (Table S1). Magnetic capsule endoscopy offers diagnostic accuracy comparable to that of standard gastroscopy while significantly reducing discomfort levels [29–31]. Regarding targeted drug delivery applications, the potential of this approach has been verified by *in vitro* studies [32–36], *ex vivo* investigations [8,37–40] and *in vivo* experiments [41]. The side effects can be effectively reduced by implementing multidrug combination therapies. Furthermore, the release of drugs at multiple target sites is beneficial, particularly for treating diseases characterized by multiple lesions, such as Crohn’s disease [42]. Hence, researchers have proposed capsule robots loaded with various drugs [34,39–41]. In addition to drug delivery, biopsies are required for diagnosis or drug evaluation in the GI environment of the human body. Typical approaches involve manipulating embedded needles [43,44] or gripping mechanisms [45,46] to collect a small fragment of the target tissue, which exhibits the risk of causing potential damage to the epithelium. Alternatively, magnetic capsule robots have been developed to sample mucus by minimizing tissue damage [41,47–51], offering a promising solution for diagnosing and treating GI diseases.

Unfortunately, the development of multichamber capsule robots with drug delivery and liquid sampling capabilities for simultaneous diagnosis and treatment of GI tract diseases is in the very early stages, restricting the better operation of capsule robots. A multichamber capsule robot is desirable throughout diagnosis and treatment to selectively complete body liquid sampling and drug release on a lesion, avoiding separate diagnosis and treatment via different methods and thereby reducing physical and psychological burdens on patients. Among the magnetic capsule robots with multiple chambers (Table S1), those with soft (or compliant) valves [40,41] exhibit simpler structures and assemblies. However, the existing dual-chamber magnetic capsule robot [41] was less flexible, as the valves could only be triggered sequentially. As a result, the valves in the earlier sequence would open simultaneously when the valves in the later sequence opened. Therefore, the liquid samples from different chambers could be mixed in the task of multiple-site liquid sampling. In addition, the previous four-chamber magnetic capsule robot [40] strongly relied on a fully liquid-filled environment and provided a low output rate, which was targeted at drug delivery only. These limitations restrict their ability to dispense medication and perform biopsy simultaneously in the GI tract via one-time oral intake. The available capsule robots provide minimal improvement in multifunctional operation, mainly because of the conflict between reducing the manufacturing complexity and increasing the functionality. Therefore, realizing a multichamber capsule robot with integrated drug delivery and liquid sampling functions is vital to the minimally invasive diagnosis and treatment of GI diseases.

Herein, we report an enhanced multichamber magnetic capsule robot (macabot) based on compliant mechanisms and programmable magnetic field actuation for selective drug delivery and liquid sampling in GI tract environments. The macabot exhibits the advantage of a simple fabrication and assembly process. It features specially designed multiple embedded modular magnetic compliant valves with different directional stiffness properties. For each chamber, the stiffness in the triggering direction is much lower than that in other directions, which enables the selective opening of individual chambers among multiple chambers by a gradient magnetic field oriented in its unique triggering direction. This allows the multiple chambers of the capsule robot to selectively execute either liquid sampling or drug releasing, which can integrate biopsy and medication modalities by one-time oral intake. Moreover, based on the multichamber design, this work proposes an *in situ* forming approach to generate a shape-adaptive hydrogel patch for targeted local treatment via sustained drug release. Rolling locomotion of the macabot in an *ex vivo* porcine stomach was implemented by a rotating magnetic field, and liquid sampling and release were demonstrated by an oscillating gradient magnet field under ultrasound imaging guidance. This work provides a method to realize both diagnosis and treatment operations, thus augmenting capsule robot functionality by enabling the selective opening of multiple chambers in a single capsule robot, and reveals the potential for precise treatment of GI diseases.

## RESULTS AND DISCUSSION

### Mechanical design and working principle of the macabot

**Figure 1.**
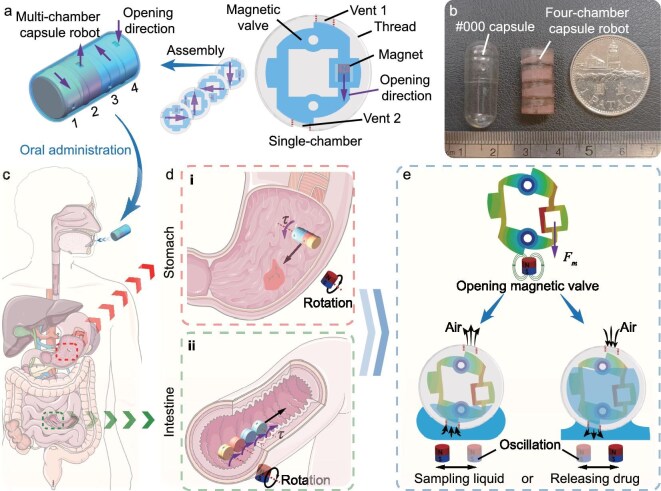
Illustration of mechanical design and workflow of the macabot. (a) Schematic of a multichamber capsule robot with outer thread by modular design of four single-chamber capsules with decoupled opening directions, which are 90$^{\circ }$ apart in one cycle. Each single-chamber module involves a magnetic valve constructed by a compliant mechanism and an embedded tiny cylinder magnet. The valve is driven by a gradient magnetic field to open vent 1 and vent 2 for sampling or release operations. Note that the four tiny magnets have different mounting directions in the four single-chamber modules. Purple arrows indicate the opening directions of four chambers. (b) Photograph of the fabricated macabot that can be capsulated inside a $\#$000 capsule for oral administration. (c) Schematic of a macabot navigating the digestive tract after oral administration. (d) Schematic of macabot rolling navigation in (i) the stomach by integrated mode and (ii) the intestine by split mode. The transition from the integrated mode to the split mode is enabled by pH-sensitive glues (e.g. shellac), which diminish the adhesion in environments with elevated pH levels. (e) Schematic showing the macabot sampling a liquid and releasing a drug by opening its magnetic valve using a gradient magnetic field, which is produced by a linearly oscillating magnet. Here $F_m$ denotes the magnetic force produced by the gradient magnetic field. Parts of (c) and (d) were produced using images from Smart Servier Medical Art (CC BY 4.0).

The macabot comprises several single-chamber capsule robots (Fig. 1a). Each one is designed with a specific opening direction by a magnetic force. Each single-chamber capsule robot has a shell with an outer thread, two vents and a magnetic valve embedded with a tiny permanent magnet ($\Phi$1.5 $\times$ 2.0 mm). The outer thread of the capsule shell is adopted to facilitate mucus clearing and to enhance the drug administration procedure [51]. Notably, to avoid sampling (or release) failure caused by air pressure, the upper vent is utilized as the inlet (or outlet) for air, thereby facilitating the flow of drugs (or bodily fluids). To enable these functions, the magnetic valve is designed as a compliant mechanism based on a four-bar parallelogram, which has the smallest vertical-to-horizontal stiffness ratio among the four design candidates, as shown in Fig. S1. The middle section for each of the two relatively long bars (top and bottom sides of the parallelogram; Fig. 1a) is perforated with a circular hole to fix the position of the magnetic valve and enable opposite moving directions of the two shorter bars (left and right sides of the parallelogram; Fig. 1a). A tiny permanent magnet is inserted into one of the two relatively short bars to be responsive to the external magnetic field.

**Figure 2.**
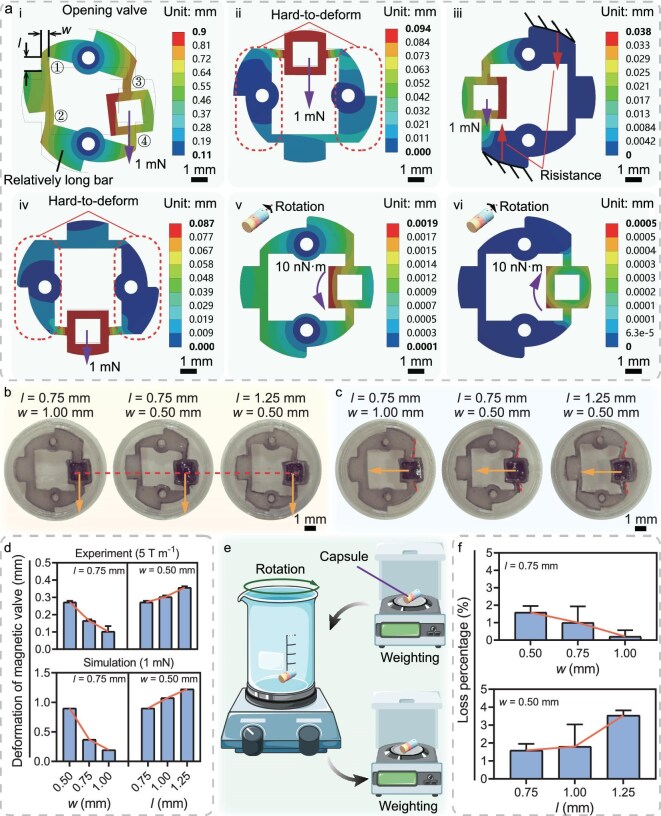
Deformation simulation and sealing test results for the magnetic valve. (a) Simulation results of the deformation distribution for the magnetic valves in (i) the opening chamber under magnetic force, (ii)–(iv) other non-opening chambers under magnetic force and (v)–(vi) the non-opening chambers under forward or backward rolling locomotion, revealing the blocking states of the magnetic valves under applied magnetic force/torque when the capsule shell is considered stationary. (b, c) Snapshots of magnetic valves with various flexure hinges under a magnetic gradient of 5 T/m applied (b) along and (c) vertical to the opening direction. The orange arrows indicate the directions of the magnetic force. The red dashed line marks the reference positions. (d) Experimental and simulation results showing the relationship between the deformation of magnetic valves and flexure hinge parameters under a specific magnetic gradient/force, indicating that the change tendencies of the experimental and simulation results are consistent. (e) Schematic of the sealing performance test by weighing the capsule before and after a 5-min rotation at a speed of 500 rpm. (f) Experimental results showing the loss percentage of macabots composed of magnetic valves with various flexure hinges, revealing a fine sealing capability of the macabots. Parts of (e) were produced using images from Smart Servier Medical Art (CC BY 4.0).

Figure 2a illustrates the deformations of the magnetic valves in the four chambers under magnetic force, and the deformations of the magnetic valves under rolling locomotion. When a magnetic force is applied in the designated opening direction of the chamber concerned, the magnetic valve undergoes an elastic deformation due to its relatively low stiffness in this direction, and the constraint from the substrate (preventing the robot’s translation). The relatively long bars rotate around the two fixing holes in a clockwise direction, allowing them to leave the two vents (for opening) on the capsule shell (Fig. 2a(i)). Otherwise, if the magnetic force is opposed to the opening direction, the capsule shell will prevent the deformation of the magnetic valve. This resistance arises from the shape and structural characteristics in that the relatively long bars cannot rotate around the two fixing holes in the counterclockwise direction (Fig. 2a(iii)). Then, suppose that the magnetic force is perpendicular to the direction of the opening. In that case, the flexure hinges close to the embedded permanent magnet deform, but the relatively long bars will remain in their original shape as they are hard to deform (Fig. 2a(ii) and (iv)). Furthermore, under the rolling locomotion, the magnetic torque from the embedded permanent magnets will be transmitted to the capsule shell as the driving power. Then, Fig. 2a(v) and (vi) demonstrate that the magnetic valves can remain in their original shape under the magnetic torques in both the clockwise and counterclockwise directions, thanks to their sufficient rotational stiffness. Hence, the magnetic force can open/close each chamber in a selective direction, revealing sufficient capability of decoupling the activation. The chambers cannot be accidentally opened during movement.

The primary structure of the magnetic valve and the associated magnet-insertion space are fabricated via laser cutting of a rubber sheet with a thickness of 1.5 mm. The shell comprises a cap and a main body manufactured via three-dimensional (3D) printing techniques. An enlarged view of a single-chamber capsule robot is shown in Fig. S2, which illustrates the spatial relationships among various components. Only simple insertion processes are required to integrate the magnetic valve into the shell and to assemble two parts of the shell. Furthermore, gluing is employed to increase the strength of connections when other components are assembled. The fabricated macabot with four chambers is illustrated in Fig. 1b, which has a size of $\Phi 9.8\, \rm {mm} \times 20.0$ mm (smaller than the $\#$000 capsule size of $\Phi 9.91\, \rm {mm} \times 26.14$ mm). Notably, the size and number of chambers can be flexibly adjusted according to practical requirements.

Figure 1c–e illustrate the working processes of the reported macabot in the digestive tract. As shown in Fig. 1c, the macabot is ingested orally, followed by bodily fluid sampling or drug release in the stomach or intestines and, ultimately, excretion through the anus. The movement of the macabot should be controlled when navigating to the target site. By applying a rotating magnetic field, the macabot can roll in the desired direction in the GI tracts, such as the stomach (Fig. 1d(i)) and intestine (Fig. 1d(ii)). In addition, the connections between the chambers can be realized by pH-sensitive glue (e.g. shellac). The chambers can be subsequently disintegrated by environments with specified higher pH values to achieve sufficient mobility in relatively narrow tracts (Fig. 1d(ii)). After arriving at the target site, the magnetic valve can be selectively actuated by applying a gradient magnetic field oriented in the corresponding triggering direction (Fig. 1e). Consequently, the two vents in the shell are opened to execute the specified operation of the open chamber, which involves either sampling the bodily fluids or releasing the drugs at the target site. The two vents are sealed by deactivating the gradient magnetic field after completing the established process. Moreover, during the execution of the specified procedure, the macabot can be actuated to oscillate linearly to enhance its interaction with the surrounding environment for better medical use.

### Characterization of the selective opening and sealing performance

The primary function of the macabot is the selective opening of each chamber, which directly ensures the efficacy of sampling liquids or releasing drugs at multiple target sites. To ensure the performance of selective opening, we investigated the elastic deformations of magnetic valves with different flexure hinges under various gradient magnetic fields. Simulation studies were carried out to evaluate the deformation distributions of magnetic valves under different triggering directions of magnetic force. Figure 2a(i) shows that the magnetic valve can only deform to open the vents when the direction of the magnetic force is aligned with the defined opening (triggering) direction. Conversely, with other conditions, the vents remain closed (Fig. 2a(ii)–(iv)). This ensures that the chamber will not open due to the activation of other chambers, which decouples the actuation of multiple chambers. For demonstration, panels (b) and (c) of Fig. 2 give snapshots of the magnetic valves with various flexure hinges under a gradient magnetic field in parallel and perpendicular directions relative to the opening direction. Figures S3 and S4 give snapshots of the magnetic valves under various gradient magnetic fields, indicating that the deformation of the magnetic valve increases with the magnitude of the gradient magnetic field (Fig. S5). In addition, the magnetic field gradients were measured at the same position. Nevertheless, the practical position of the embedded magnet must be closer to the external permanent magnet as the deformation of the magnetic valve increases. Therefore, the curve in Fig. S5 shows a nonlinear feature. To examine the influence of flexure hinges on opening performance, the deformations of magnetic valves with various flexure hinges are quantified via simulation and experimental results, as shown in Fig. 2d. By reducing the in-plane width (*w*) or increasing the in-plane length (*l*) of the flexure hinge (Fig. 2a), the deformation of the magnetic valves increases (Fig. 2d). This finding indicates that the ease of opening the presented magnetic valve can be adjusted by tuning the stiffness of the flexure hinges. Furthermore, the tendencies observed in the simulation and experimental results exhibit a high degree of consistency, demonstrating that a preliminary evaluation can be conducted via a simulation study to evaluate the robot’s performance. Hence, to investigate the influence of physical force on the GI tract, the pressure applied by the four-chamber macabot to the substrate was measured under a magnetic gradient of 5 T/m. The measurement result indicates a force of 3.8 mN, equivalent to the gravitational weight of approximately 0.39 g of food. If considering the contact area as 51.29 mm$^2$ (one-sixth of the cylindrical surface area), the pressure is derived as 74.09 Pa, which is much smaller than the mean inflation pressure during the common gastroscopy (1.33 kPa) [52]. Therefore, the macabot is safe for the patient. Otherwise, the forces from the simulation and experimental results are similar, but with a significant difference in deformation. This may result from the friction of the inclusion, according to Equation (S8). In addition, with a magnetic gradient of 5 T/m, the distance between the macabot and the employed permanent magnet is 3.1 cm. For targeting deeper sites, it is advisable to utilize either a larger permanent magnet or a superconducting coil; such devices have already been commercialized and are now available as products [53].

During the navigation of the macabot through the digestive tract, the sealing capabilities play a crucial role, as they govern whether the enclosed drugs or sampled bodily fluids are accidentally released during the patient’s physiological activity. To investigate the sealing performance, single-chamber capsule robots with various flexure hinges were placed in an empty container with a rotation speed of 500 rpm for 5 min. The weights of the capsule robots before and after rotation were measured to calculate the percentage of loss (Fig. 2e). The rotation speed of 500 rpm is significantly more intense than the regular physiological activity in the GI tract [54]. Figure 2f depicts the sealing performance (i.e. loss percentage) of capsule robots composed of magnetic valves with various flexure hinges. The variation tendency of the data in Fig. 2f is similar to that in Fig. 2d as the length *l* and the width *w* of the flexure hinges change, which indicates that the loss percentage is correlated with the ease of opening the magnetic valve. Specifically, the smaller the elastic deformation (i.e. the higher the flexure hinge stiffness), the smaller the loss percentage of the enclosed liquid. This results from the fact that compliant mechanisms with high stiffness make it difficult to induce large oscillations due to their relatively high resonant frequencies, thereby preventing the enclosed liquids from escaping through the vents. Using flexure hinges with *l* = 0.75 mm and *w* = 0.50 mm, the average loss percentage of the enclosed liquids is lower than 2%, which is adequate for practical applications. Hence, magnetic valves with such dimensions were adopted for subsequent studies.

### Locomotion and controllability capacities of the macabot

**Figure 3.**
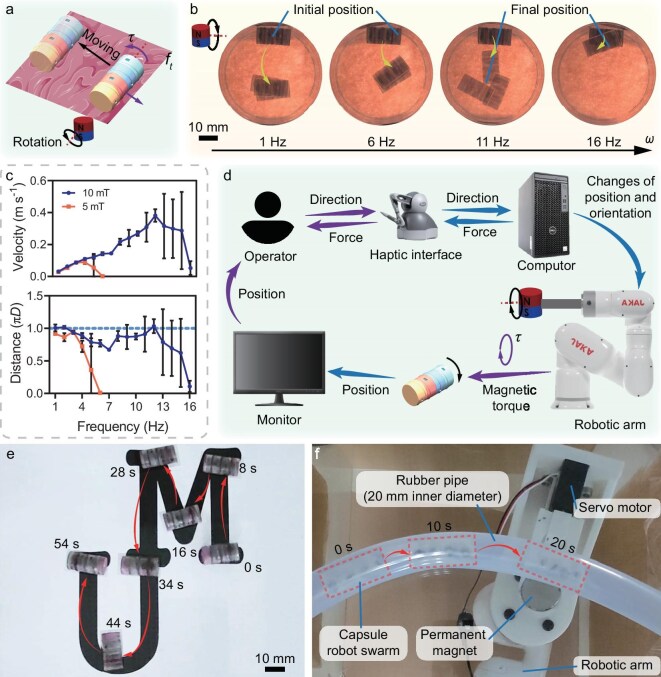
Locomotion and controllability test results of the macabot. (a) Schematic of the locomotion mode of the macabot by surface rolling enabled by a rotating magnetic field. The magnetic torque ($\tau$) is generated to propel the macabot to roll forward by overcoming the friction ($f_t$). (b) Snapshots of three movement trials of the macabot under a uniform rotating magnetic field of 10-mT magnitude and various rotating frequencies. The yellow arrows indicate the moving directions of the macabot. (c) Locomotion velocity and distance of the macabot versus the rotating frequency of the magnetic field with 5- and 10-mT magnitudes. Here *D* is the diameter of the macabot. The used programmable rotating magnetic field is provided by the 3D Helmholtz coils depicted in Fig. S10. (d) Schematic of the hardware connection of a custom-built teleoperated robotic system adopted for locomotion experimental studies. (e) Snapshots of the capsule robot tracking along the ‘UM’ shaped path by integrated locomotion mode, extracted from Movie S1. (f) Snapshots of the capsule robot swarm navigating inside a rubber pipe with an inner diameter of 20 mm by split locomotion mode, extracted from Movie S1. Parts of (a) and (d) were produced using images from Smart Servier Medical Art (CC BY 4.0).

To achieve active navigation to the target site, the locomotion and controllability capacities of capsule robots are crucial. Figure 3a shows a schematic diagram of the macabot locomotion mode. Under a uniform rotating magnetic field, a magnetic torque ($\tau$) is generated by the directional difference between the embedded permanent magnets and the external magnetic field, which causes rotation of the macabot. Moreover, the robot is propelled forward by overcoming the friction ($f_t$) between the macabot and its contact surface.

To investigate the locomotion capacities, the macabot was actuated by a uniform rotating magnetic field with a magnitude of 5 and 10 mT at various rotating frequencies ($\omega$) within a single cycle. The initial and final positions of the macabot at 1–16-Hz rotating frequencies (each with three trials) are shown in Fig. 3b. With a magnitude of 10 mT, the movement of the macabot becomes unstable as the rotating frequency increases, ultimately ceasing at a higher frequency of 16 Hz. It is observed that there are deviations in the locomotion of the macabot at 6 and 11 Hz. This might be caused by the unilateral stick-slip motion of the unsmooth rolling. Under a different magnetic field (5 mT), the velocities and distances of the macabot were obtained, as shown in Fig. 3c. The results indicate that the macabot achieves the maximum speed (0.38 m/s) under a magnetic field of 10 mT with a rotating frequency of 12 Hz. However, the standard deviation (SD) of the data at 12 Hz is relatively high, indicating that the movement is unstable. The results of the moving distance further demonstrate this phenomenon. The moving distance closer to the perimeter of the macabot suggests that the rolling of the macabot better follows the step of the rotating magnetic field. In contrast, the macabot is out of step with the rotating magnetic field. Therefore, under a magnetic field with a rotating frequency lower than 2 Hz, the movement of the macabot is stable as the moving distance is closer to the perimeter with a slight SD. Moreover, it is observed that the stability of the macabot under a magnetic field of 10 mT is larger than that under a magnetic field of 5 mT, indicating that a stronger magnetic field could effectively support the macabot to move stably. For information, the peristaltic (contraction) wave of the GI tract travels at speeds of 2–20 mm/s, and the peristaltic wave of the GI tract is conducted approximately 3–10 times per minute. Therefore, the locomotion speed of the macabot is sufficient to precisely deploy to the target site under the GI peristaltic since the macabot can achieve a speed of 62.6 mm/s at 2-Hz rotating frequency. However, the impact of intestinal peristalsis on the accuracy and stability of capsule operations requires further investigation, which may be compensated for by an advanced control algorithm. This is not the primary focus of this article. Such work will be incorporated into our future studies.

The macabot can locomote in the integrated or split mode. Given the distinct pH environments within the GI tract, a pH-sensitive glue (with diminished adhesion at elevated pH levels) enables the macabot to transition into a split mode as it traverses from the stomach to the intestine. To evaluate the controllability of the macabots, we conducted navigation experiments under two distinct states, i.e. integrated assembly and split individual units, imitating navigation in gastric and intestinal scenarios, respectively. The navigation process was enabled by a custom-designed teleoperated robotic system that regulated the rolling direction of the macabots (Fig. 3d). In the integrated mode, the four-chamber capsule robot successfully traced a complex ‘UM’ trajectory (Fig. 3e and Movie S1). Alternatively, the split mode enabled parallel navigation of four independent single-chamber units in a rubber pipe with an inner diameter of 20 mm (Fig. 3f and Movie S1). These results verify that the macabot possesses remarkable controllability and operational flexibility in both integrated and split locomotion modes.

### Characterization of the liquid sampling performance

**Figure 4.**
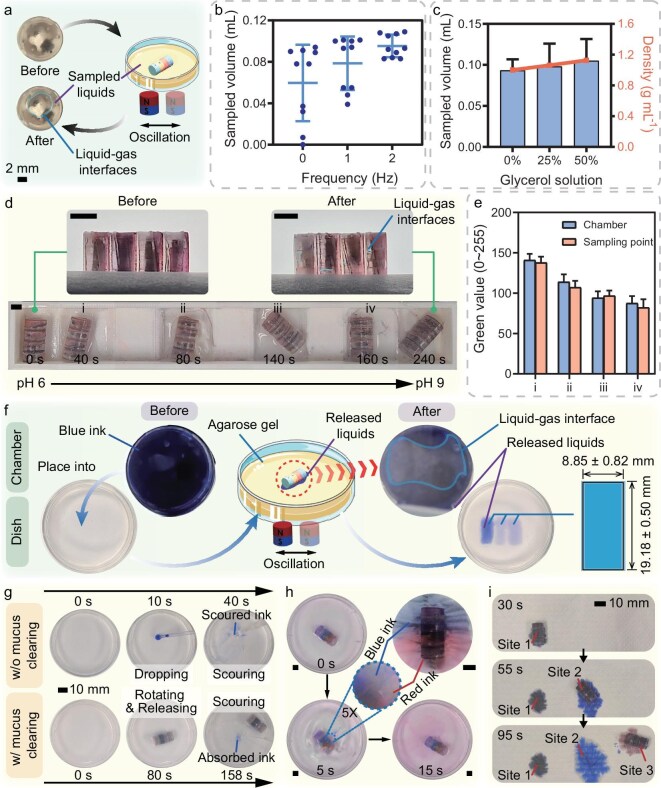
Liquid sampling and release test results of the macabot. (a) Schematic of liquid sampling achieved by linearly oscillating the magnet. (b) Relationship between the sampled volume and the oscillating frequency of the external magnet, indicating that the magnet’s oscillation can promote the fluid flow to increase the sampled volume. (c) Relationship between the sampled volume and the density of the liquids, indicating that the sampled volume increases with increasing liquid density. (d) Snapshots of the sampling test at four points (i)–(iv) with gradually increased pH values, extracted from Movie S2. Scale bars are 5 mm. (e) Green values of the pH test papers measured from the liquids in the chambers and corresponding sampling points (i)–(iv). The consistency between the two results demonstrates the effectiveness of the separate sampling operation. (f) Schematic of releasing blue inks in the agarose gel by linearly oscillating the external magnet. The inks were released on three rectangles with similar magnitudes, demonstrating the macabot’s robust reproducibility for liquid release. (g) Anchoring results of released inks with and without mucus clearing, showing that the macabot enhanced the drug diffusion into agarose gel at the bottom of the Petri dish by crossing the mucus barrier on the surface. (h) Snapshots of simultaneously releasing the encapsulated contents from two chambers with blue and red inks, extracted from Movie S3. Scale bars are 5 mm. (i) Three-chamber capsule robot for selectively releasing different encapsulated contents at three sites in sequence (site 1, black ink; site 2, blue ink; site 3, red ink) extracted from Movie S3, indicating the effectiveness of multiple-site liquid release on demand. Parts of (a) and (f) were produced using images from Smart Servier Medical Art (CC BY 4.0).

Next, we investigated the liquid sampling capabilities of the macabot to determine the maximum sampling volumes achieved in various liquids. As illustrated in Fig. 1e, liquid sampling is enabled by the fluid flow induced by the pressure difference between the upper and bottom vents. As shown in Fig. S6A, the pressure distributions of an empty macabot for the sampling liquid are illustrated by a simulation study, which verifies the effectiveness of the sampling induced by the pressure difference. To extend the investigation, the empty capsule robot was positioned in a Petri dish filled with liquids of various densities (Fig. 4a). The capsule robot was submerged in the liquids with the depth close to the upper vent, thereby maximizing the pressure differences conducive to effective sampling. Notably, the actuating permanent magnet provided an average magnetic field gradient of 5 T/m to open the magnetic valve. The magnet oscillated linearly to promote fluid flow. Photographs of the macabot before and after sampling are displayed in Fig. 4a. The liquids were successfully sampled under the actuation of the actuating permanent magnet. However, a liquid–gas interface (air bubble) in the sampling chamber indicated that the chamber was not fully filled with liquid. This was due to an insufficient pressure difference to push the bubble out of the upper vent. To investigate the influence of the linear oscillation of the magnet, the weights of the macabot were recorded before and after 10 times of sampling, while the magnet oscillated with an amplitude of 1 cm at various frequencies. Figure 4b shows the sampled volumes under various frequencies, revealing that the probability of successful sampling (sampled volume above 0.08 mL) increases with oscillating frequency. This result demonstrates that oscillation can effectively enhance the fluid flow. Moreover, the weights of the macabot before and after sampling were recorded to investigate the sampling performance for liquids of various densities. The magnet oscillated at a frequency of 2 Hz with an amplitude of 1 cm. The sampled volumes are calculated and summarized in Fig. 4c, indicating that the sampled volume increases with the density of the sampled liquid. This may be induced by the increasing pressure difference as the density increases. In addition, a single chamber of the capsule robot can sample approximately half the inside volume of a single chamber.

To sample liquids at multiple target sites without the risk of unintended mixing, it is essential to ensure that subsequent samples do not affect those taken from previous chambers. A macabot was employed to sample the liquids in four pits via remote control to demonstrate this function. Each pit has a depth of 2 mm (Fig. 4d and Movie S2). Since the liquid depths were limited, paper scraps were placed into the chambers. The water absorption of the paper can effectively increase the volume of sampled liquids under situations with limited liquid depth. Additionally, the liquids in the four pits had different pH values for further evaluation of the mixture conditions of the sampled liquids. After multisite sampling, the liquid–gas interfaces in the chambers demonstrated successful liquid sampling (Fig. 4d). Figure 4e and Fig. S7 show the comparison between the pH values of the liquids in the chambers and the sampling points. The results indicate that the pH values remain highly consistent for the liquids from the chambers and the corresponding sampling points. These results demonstrate the effectiveness of the macabot method for sampling liquids from different target sites separately.

### Characterization of liquid-releasing performance for targeted therapy

We subsequently investigated the resolution of the macabot release area at the target site. As illustrated in Fig. 1e, the difference in the liquid pressure between the capsule inclusion and the surroundings allows the enclosed drug to be released into the surroundings. Figure S6B within the online supplementary material shows simulation results illustrating the pressure distributions of a fully filled macabot during liquid release, which verifies the effectiveness of release induced by the pressure difference. Moreover, the capsule robot containing blue ink was positioned in a Petri dish filled with agarose gel (Fig. 4f). The agarose gel has great water absorption ability, which enables the released blue ink to stay at the release site for better observation. In addition, the actuating permanent magnet provided the same magnetic gradient field as the liquid sampling test (Fig. 4a). The release operation was conducted three times, each within 5 min. Figure 4f shows the procedure for macabot release of the enclosed blue ink. The release area did not noticeably change. Therefore, considering the release site with high water absorption, the average release area is $19.18 \times 8.85$ mm$^2$, which is slightly smaller than the projected area of the capsule robot. Moreover, the light ink prints indicated that the ink had been absorbed into the deep layer of the agarose gel. These results suggest that the drug released from the capsule robot to the release site would diffuse with the contact surface. Furthermore, the results reveal small differences among three release areas, demonstrating the macabot’s robust reproducibility under controlled experimental conditions. Notably, if the surrounding material is liquid, the released drugs may be restricted (or diffused) by the liquid, increasing (or reducing) the position resolution of the release area (Fig. 4g and h). According to the Stokes–Einstein relation, high-viscosity liquids reduce the diffusion speed of drugs.

In practice, a layer of mucus is present on the surface of the intestines and is characterized by its periodic self-clearing properties. Consequently, if the released drug cannot penetrate the mucus barrier in time, its bioavailability decreases as mucus clearance clears the drug. A practical solution involves removing the mucus layer, after which the capsule releases the drug under the mucus barrier. To demonstrate its efficacy, we compared the ink release results with and without mucus clearing (Fig. 4g and Movie S3). In addition, the mucus was modified with a 1.2% sodium alginate solution [47]. Without mucus clearing, the ink was dropped onto the upper mucus layer, and then the water could scour it away since the bottom agarose gel did not absorb it. In contrast, with mucus clearing, by actuating the capsule robot to rotate and squeeze out the mucus, the ink was directly released and absorbed by the bottom agarose gel, which could not be scoured away. These results indicate that the capsule robot can directly release the drug under the mucus barrier, increasing drug bioavailability.

Furthermore, the opening directions of the chambers can be reconfigured in the same direction. This design enables separate storage of various contents within different chambers, avoiding the loss of efficacy due to premature reactions. Consequently, the encapsulated contents can be selectively released at the target site, allowing for mixing operations after release. This approach can be applicable to some instances involving combination drug therapy. Figure 4h and Movie S3 show the capsule robot setup for selectively releasing the encapsulated contents from two chambers. The inks with different colors are released from the two chambers, which are then mixed in the water. Next, by reducing the number of chambers, the volume of each chamber can be appropriately increased. According to the dimensions of various parts of the digestive tract, it is recommended that the length and diameter of the chambers remain smaller than the diameter of a $\#$000 capsule. For demonstration, a three-chamber capsule robot with a dimension of $\Phi 9.8 \times 6.5$-mm was fabricated to experimentally study the release of inks of different colors at three sites (Fig. 4(i) and Movie S3). The results verified the effectiveness of the macabot configuration with fewer chambers and larger volume, as well as the ability to selectively release drugs at multiple target sites. In addition, it is observed that there are instances of blue ink leakage occurring between site 2 and site 3. As shown in Movie S3, the leakage occurred when the vent of the corresponding chamber made contact with the substrate again. Moreover, the leakage area is around 1.66% of the ink area in site 2, which is a relatively low magnitude. Therefore, these leakages may primarily originate from the internal liquid expelled by the magnetic valve toward the vents during the structural reset process. Afterward, the leakages adhere to the capsule surface and are absorbed by the adjacent tissue during the rolling locomotion. Thus, it can be deduced that the absorbing area is close to the last releasing site, exerting a minimal effect. Such leakages can be mitigated by adding a stop period immediately following the release operation at the target site. Then, the expelled liquid will be absorbed by the release site, as exemplified by the procedure at site 1.

Except for the instantaneous release of the drug, the macabot can achieve sustained release of the drug by *in situ* forming a hydrogel patch at the target site. This will benefit the treatment of chronic diseases in the GI tract, such as IBD. In this study, we selected the commonly used sodium alginate-based hydrogel for demonstration. Its primary polymer and crosslinker are 0.5% sodium alginate solution and 0.5% calcium chloride (CaCl$_2$) solution. As shown in Fig. S8, the macabot is allowed to store the sodium alginate solution and CaCl$_2$ solution within different chambers. In addition, the drug can be mixed into the sodium alginate solution. Then, a shape-adaptive hydrogel patch is formed *in situ* at the target site by releasing the sodium alginate solution and CaCl$_2$ solution in sequence, and then the enclosed drug is released sustainably. Figure S8C shows the released mass of the fluorescein isothiocyanate (FITC) loaded in a hydrogel patch over time, demonstrating the effectiveness of the sustained release. There was approximately 1 $\mu{\rm g}$ of released mass at the 0-h time instance, which was due to the high diffusivity of the FITC. Before the formation of the hydrogel patch, the FITC had already diffused into the excess CaCl$_2$ solution. Thus, it can be mitigated by reducing the residual liquid after patch formation. Notably, the initial drug concentration may not be disadvantageous for some scenarios. Moreover, we further studied the release with the reverse sequence. It was found that the hydrogel would be formed inside the chamber, as shown in Fig. S9. It may result from the Ca$^{2+}$ ions rapidly entering the chamber enclosed with the sodium alginate solution by liquid diffusion.

### *Ex vivo* experimental studies of liquid sampling and release in the porcine stomach

**Figure 5.**
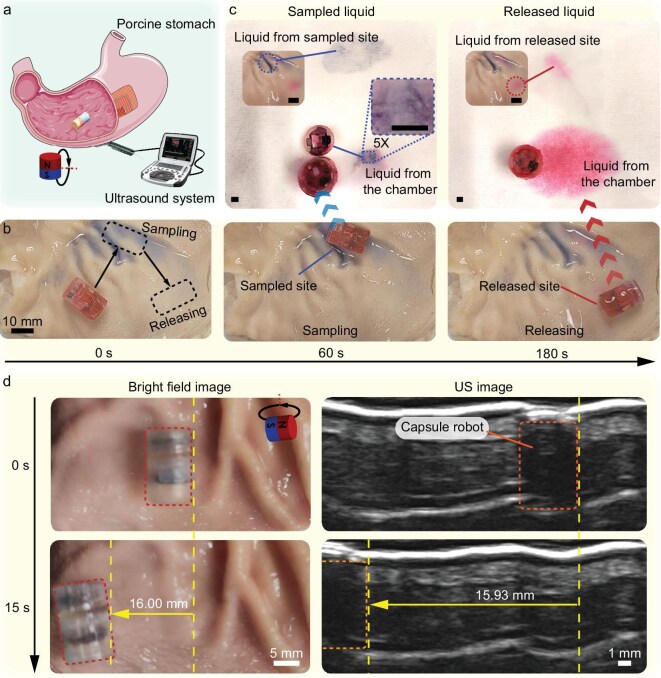
*Ex vivo* test results in the porcine stomach. (a) Schematic of the *ex vivo* test setup under the guide of a US imaging system. (b) Snapshots of a dual-chamber macabot for executing biopsy and medication by liquid sampling and release at different sites in sequence, extracted from Movie S4. (c) Mock results of liquid sampling and release operations for biopsy and medication applications. Scale bars are 2 mm. (d) Bright-field images and US images of the capsule robot navigation in the porcine stomach by rolling locomotion, demonstrating the effectiveness of *in vivo* navigation under US imaging. US images are extracted from Movie S4. Parts of (a) were produced using images from Smart Servier Medical Art (CC BY 4.0).

To demonstrate the effectiveness of the robot under biologically relevant environments, we tested the liquid sampling and release functions of the reported macabot in an *ex vivo* porcine stomach, as illustrated in Fig. 5a. Furthermore, *in vivo* imaging is crucial for stably guiding the motion of the capsule robot. Ultrasound (US) imaging has several advantages, including its noninvasive nature, cost effectiveness, ability for real-time visualization, high portability and lack of radiation. Hence, it is suggested as an imaging approach for practical applications. Its effectiveness is verified in this study.

For demonstration, a dual-chamber capsule robot was fabricated to conduct an experimental study of liquid sampling and release of the encapsulated content at different sites (Fig. 5b and Movie S4). To illustrate the flexibility of the capsule robot design, the dimensions of the two single chambers for sampling and releasing were designed as $\Phi 9.8\,\rm {mm} \times 5$ and $\Phi 9.8\,\rm {mm} \times 10$ mm, respectively. The sampled liquid and released liquid were subsequently extracted for comparison with their original liquids (Fig. 5c), demonstrating the precision of the capsule robot for selective sampling and releasing operations and the effectiveness of integrated biopsy and medication via one-time oral intake. Notably, such functions cannot be implemented by existing capsule robots

Figure 5d shows a series of snapshots (extracted from Movie S4) of the US image for actuating the capsule robot to navigate an *ex vivo* porcine stomach. The boundary between the macabot stomach and the porcine stomach exhibits corresponding displacement during navigation. Moreover, the displacements of the capsule robot are derived from bright-field images and US images, which remain consistent. These results verify the effectiveness of using US imaging to guide the operation of the reported capsule robot. Taken together, the *ex vivo* tests validated the selective liquid sampling and release capability of the macabot in biologically relevant environments.

## CONCLUSIONS

This article proposes a new design for a capsule robot with the functions of multichamber selective opening, targeted bodily fluid sampling and on-demand drug release. The selective chamber opening mechanism is achieved by an embedded compliant mechanism that responds exclusively to a unidirectional magnetic force. This design allows the capsule robot to navigate to target locations via a uniform rotating magnetic field, followed by liquid sampling or drug-releasing operations controlled by a directional gradient magnetic field. This approach demonstrates significant clinical potential by enabling progressive biopsy and drug delivery, as well as multiple sites or times of biofluid sampling and medication, all through one-time oral intake, thereby potentially increasing patient comfort and procedural efficiency.

To systematically optimize the structure of the magnetic valve, we investigated the influence of its structural parameters on both its activation performance and sealing capability. *In vitro* experimental studies demonstrated the reported capsule robot’s navigation, sampling and releasing capabilities. Furthermore, we explored various chamber configurations with multiple sizes and quantities (2, 3 or 4) in experimental studies, validating the adaptability of the modular design to diverse requirements. Subsequently, *ex vivo* experimental studies verified the effectiveness of the reported capsule robot under US imaging in biologically relevant environments. These results confirm that the macabot is a promising tool for performing biopsies and delivering medication at multiple sites or at different times. Based on the selective opening/closing of multiple chambers with solutions preloaded, the work demonstrated *in situ* generation of a shape-adaptive hydrogel patch for localized treatment, providing a promising solution for sustained drug release to treat GI diseases.

The number of chambers in the macabot can expand to more than four by introducing predeformation to the magnetic valve. The predeformation of the magnetic valve can be achieved by specially designing the shape of the inner wall. The underlying mechanism relies on the principle that, when the magnetic force component in its triggering force is below the predeformation-induced restoring force, the magnetic valve will not be activated, resulting in insensitivity to the vertical magnetic force. Therefore, by increasing the resolution of the activating direction, we can integrate a larger number of chambers (e.g. more than eight) within a single capsule robot while maintaining the function of selective opening. However, it increases the required magnetic force for opening the chamber. Hence, the design for implementing such an expansion should be carefully evaluated according to the task requirements. Furthermore, due to the magnetization of the macabot, the capsule robot is limited to five degrees of freedom (DOF) in its motion, indicating that it lacks complete three-dimensional spatial mobility. Therefore, to further enhance its dexterity, it is desirable to employ the anisotropic magnetization approach [40,55–57] to realize six-DOF motion, thereby achieving more locomotion modes, such as walking and swinging. Then, the integration of diverse locomotion modes can effectively enhance the robot’s ability to execute tasks within complex environments [58,59].

In addition to the GI tract, targeted diagnosis and treatment within small-diameter bodily tracts, such as the bile and pancreatic ducts, also require further research. The primary challenge in designing specialized capsule robots for these applications lies in the strict size constraints. While compliant mechanisms offer advantages in miniaturization, scaling down the capsule robot inevitably reduces the size of fluid vents, thereby increasing the difficulty of fluid interaction between the encapsulated contents and the surrounding biological fluids. Furthermore, the anticipated reduction in the magnet’s size also requires attention. For instance, with the magnet’s size of $\Phi 1\,\rm {mm} \times 0.5$ mm (the minimal size available on the market), the diameter of the macabot with the same flexure hinges can be reduced to 7.8 mm, but the required magnetic field intensity increases approximately three-fold. Consequently, it is necessary to reduce the width of the flexure hinges to achieve a lower stiffness, which presents challenges for the manufacturing process. In addition, the laser cutting machine can only produce hinges with minimal width around 0.25 mm, and the resulting hinges are prone to breakage during the laser cutting. Additionally, air inflation in the bile and pancreatic ducts could pose risks, as pressure has been linked to the pathogenesis of certain GI diseases [60,61]. Thus, further research is necessary to assess the feasibility of the reported air-based sampling method in such cases. Furthermore, the reported macabot can only release drugs through the basic liquid diffusion within a fully liquid environment, which may be slow. To broaden the applicability of capsule robots in such environments, it is desirable to explore more structural designs with actuation mechanisms (e.g. ultrasonic actuation or thermal actuation) to enhance both the initiative and versatility of liquid sampling and releasing operations [62]. Moreover, *in vivo* experiments are needed to further examine how the dynamic environment of the GI tract affects the accuracy and stability of capsule operations, as well as to assess the practical applicability of the proposed macabot.

## METHODS

### Materials and fabrication

Ecoflex-0050 (from Smooth-On, Inc.) was poured into a mold to form a rubber sheet with a thickness of 1.5 mm. Then, the primary structures were cut by a UV laser cutting machine (DBJ-UVM, from Dongguan Diaojiang Intelligent Technology Co., Ltd.). The embedded permanent magnets were N52 models with a size of $\Phi 1\,\rm {mm} \times 1.5$ mm. The capsule shells were fabricated by a 3D printer (Objet30, from Stratasys Ltd.) with VeroClear material. Unless otherwise specified, the experimental studies were conducted with a four-chamber capsule robot. In addition, the components that interface with the tissue include the magnetic valve and the capsule shell. Regarding the magnetic valve material (Ecoflex-0050), it is a biocompatible and biodegradable plastic widely used in medical devices. Furthermore, the VeroClear material of the capsule shell is chosen for its high transparency, which enhances the ability to conduct experimental observations. In practical applications, this material can be readily substituted by a biocompatible material, as it does not experience deformation during the operation.

Detailed methods are provided in the online supplementary material.

## Supplementary Material

nwaf400_Supplemental_Files
