# Supplementary material for: Multichamber magnetic capsule robot for selective liquid sampling and drug delivery
Source: Natl Sci Rev. 2025 Sep 19;12(11):nwaf400. doi: 10.1093/nsr/nwaf400 (PMC12576952; doi:10.1093/nsr/nwaf400)
Supplement: nwaf400_Supplemental_Files [file nwaf400_supplemental_files.zip › Supplementary data.pdf]

SUPPLEMENTARY MATERIALS

Multichamber magnetic capsule robot for selective liquid sampling and drug delivery

Zehao Wu[1], Xianli Wang[1], Yang Lu[2,*], and Qingsong Xu[1,*]

[1]Department of Electromechanical Engineering, Faculty of Science and Technology, University of Macau, Macau, China

[2]Department of Mechanical Engineering, The University of Hong Kong, Hong Kong, China

*Corresponding authors. Email: qsxu@um.edu.mo (Q. Xu); ylu1@hku.hk (Y. Lu)

This PDF file includes:

Supplementary Text

Figures S1 to S15

Table S1

Movies S1 to S4

Other Supplementary Materials for this manuscript include the following:

Movies S1 to S4

Supplementary Text

Section S1. Methods

Fluid simulation

The fluid simulations were conducted via COMSOL software. The enclosed fluid was determined to be air or pure water without the influence of inertia force. The inlet/outlet had a velocity field of 1 mm/s in the y-direction, and the outlet was determined with zero pressure and no backflow. All boundaries were defined as no-slip boundaries.

Magnetic actuation system

The magnetic gradient for driving the magnetic valves was provided by an N52 permanent magnet with a size of $\Phi 30 \times 30$ mm. The magnetic gradients and intensities exerted on the center of the macabot were measured by a Tesla meter (model: TLV493D-A1B6, from Infineon Technologies AG.). If not otherwise specified, the magnetic field was provided by the external permanent magnet, and the preenclosed liquids were loaded by injection through the vent. In addition, the maximum superficial magnetic field of the actuation permanent magnet is measured as 380 mT, which satisfies the safety requirement [62].

Deformation test

The simulations of the magnetic valves were carried out via static structural analysis via ANSYS Workbench software. The mesh and the setup of the static structural analysis are shown in Supplementary Fig. S10. The solver was set to consider the large deflection. The place of the embedded permanent magnet was set as nondeformable. Furthermore, the experimental tests were carried out three times, and the mean $\pm$ SD values were derived.

Sealing test

The empty container had a diameter of 55 mm. The rotation of the empty container was provided by a spin coater (model: KW-4A, from Beijing Tongshi Huagang Equipment Co. Ltd.). The experimental tests were carried out four times, and the mean $\pm$ SD values were subsequently derived.

Locomotion test

In the experimental studies shown in Figs. 3B and 3C, uniform rotating magnetic fields were produced by custom-built 3D Helmholtz coils (Supplementary Fig. S11). The input currents to the coils were regulated by servo amplifiers (model: ESCON 70/10, from Maxon Motor AG), which were controlled by a driving board (model: PCI-6259, from National Instruments Corp.) utilizing a program developed in LabVIEW software running on a personal computer.

Teleoperated robotic system

A 6-axis robotic arm (model: JAKA MiniCobo, from JAKA Robotics Co., Ltd.) with an additional servo motor at the end was adopted to manipulate the spatial position and orientation of the permanent magnet precisely. The control signal was obtained through a haptic interface (model: Geomagic Touch, from 3D Systems Corp.). The changes in position and orientation were dynamically adjusted according to the moving direction of the handle of the haptic interface. Additionally, the haptic interface also offered the operator real-time tactile resistance opposite to the direction input. This bidirectional feedback mechanism enables the precise modulation of directional inputs during the manipulation process. Supplementary Fig. S12 illustrates the teleoperated robotic system.

Liquid sampling test

The sampled liquids were prepared by mixing various ratios of pure water and glycerol (99%, from Xilong Scientific Co. Ltd.). The experimental tests with each material ratio were carried out four

times, and the mean $\pm$ SD values were derived. The passage with four pits was custom-built with 3D printing using polylactic acid (PLA) material. The structural diagram of the passage with four pits is shown in Supplementary Fig. S13. The green values of the pH test papers were determined as the mean $\pm$ SD values of the contact area with the liquids.

Release test

A 1.5% agarose solution was prepared and subsequently heated to 150°C. The heated solution was subsequently transferred to a Petri dish at room temperature, where it formed an agarose gel. Agarose agar powder and sodium alginate ($(C_6H_7NaO_6)_n$, 99.5%) were purchased from Sinopharm Chemical Reagent Co., Ltd.

Hydrogel patch test

A 0.5% sodium alginate solution and 0.5% $CaCl_2$ solution were prepared to generate the hydrogel patch. The interfaces with various shapes were custom-built with 3D printing using VeroClear material (from Stratasys Ltd.). In addition, the interfaces were spread with a little pure water to enforce the liquid release. The structural diagrams of the interfaces are shown in Supplementary Fig. S14. $CaCl_2$ (99.7%) was purchased from Sinopharm Chemical Reagent Co., Ltd, China. FITC was purchased from Aladdin Scientific Corp. The hydrogel patches were formed by using 100 µL of each solution. The released masses were determined as the mean $\pm$ SD values of three experimental results.

US imaging test

The US images were collected by the US system in 2D mode (model: Terason uSmart 3300, from Terason Division Teratech Corp.).

Section S2. Analytical modeling of the macabot

In this section, the working principle of the macabot is introduced by conducting analytical modeling. For both the locomotion of the macabot and the opening of the magnetic valve, the macabot is actuated by an external magnetic field ($\boldsymbol{B}$). The magnetic torque (τ_m) and magnetic force (F_m) exerted on each embedded permanent magnet in the macabot can be calculated as follows.

$$\tau_m = \boldsymbol{m} \times \boldsymbol{B} \tag{S1}$$

$$F_m = \boldsymbol{m} \cdot \nabla \boldsymbol{B} \tag{S2}$$

where $\boldsymbol{m}$ is the equivalent magnetic moment of the embedded permanent magnets.

As depicted in Supplementary Fig. S15A, the locomotion of the four-chamber capsule robot is assumed as stable rolling motion without slipping. The actuated torque (τ) and the resistant torque (τ_r) are determined below.

$$\tau = \tau_r \tag{S3}$$

$$\tau = 4|\boldsymbol{m}||\boldsymbol{B}|\cos(\theta(\omega)) \tag{S4}$$

$$\tau_r = f_t r = \mu T r = \mu G r \tag{S5}$$

where θ is the angle between the magnetic moment and the actuating magnetic field of the capsule robot, whose minimum value increases as the rising of the alternative frequency (ω) of the magnetic field until step-out occurs. f_t, T, and G are the friction, supporting force, and gravity, respectively. μ is the friction coefficient. r is the rolling radius. Notably, resistant torque should be remodeled for different rolling environments, e.g., considering the buoyancy and the magnetic force. In addition, concerning the step-out motion, it is defined as the motion of the capsule robot which cannot follow the rotation of the magnetic field. This is resulted due to the resistant torque is larger than the actuating torque.

Supplementary Figures S15B and S15C show the structural parameters and the scheme diagram of the magnetic valve, respectively. Under the influence of the magnetic force, the relationships between the displacement of the embedded permanent magnet (d_m) and the rotation angle of the relative long bars (α) can be derived as:

$$\cos\alpha = d_m/b \tag{S6}$$

where b denotes the equivalent length of the relatively long bars. Thus, the rotation angles of the four flexure hinges are determined as α, and their rotational stiffnesses (K) are derived based on the Euler-Bernoulli beam theory as follows.

$$K = \frac{Etw^3}{12l} \tag{S7}$$

where t, w, and l are the out-of-plane thickness, in-plane width, and in-plane length of the flexure hinges, respectively. E is the equivalent Young's modulus of the material. The magnetic gradient, the equivalent friction from the inclusion (f_d), and the friction torque from the shafts (τ_s) are assumed as constants. Therefore, based on the energy conservation law, the system of the magnetic valve can be determined as:

$$F_m d_m = 2K\alpha^2 + 2\tau_s\alpha - \Delta G_{sh} d_m - f_d d_m \tag{S8}$$

where ΔG_{sh} is the equivalent gravity difference between the two relatively short bars. In addition, the above modeling assumes that the magnetic torque and the magnetic force are completely decoupling for robot locomotion and magnetic valve opening.

Supplementary Figures

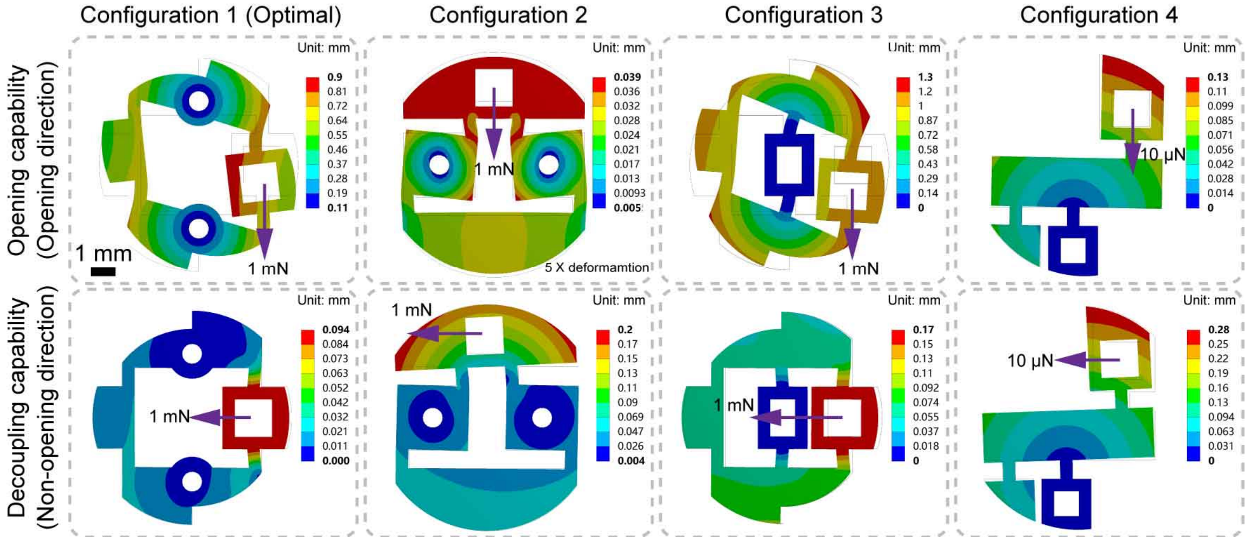

Fig. S1. Configurations of four structure design candidates of the magnetic valves. It shows the simulation results of deformation distribution for four design candidates of the magnetic valve under the magnetic force applied along and vertically to the opening directions, demonstrating the performances of selective opening for the magnetic valves.

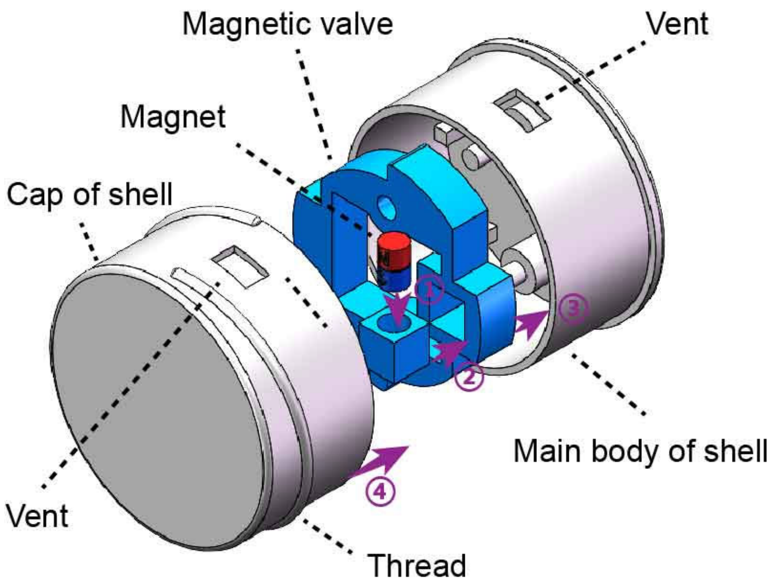

Fig. S2. Exploded view of a single-chamber capsule robot. Purple arrows: installation direction; purple numbers ①②③④: assembly sequence.

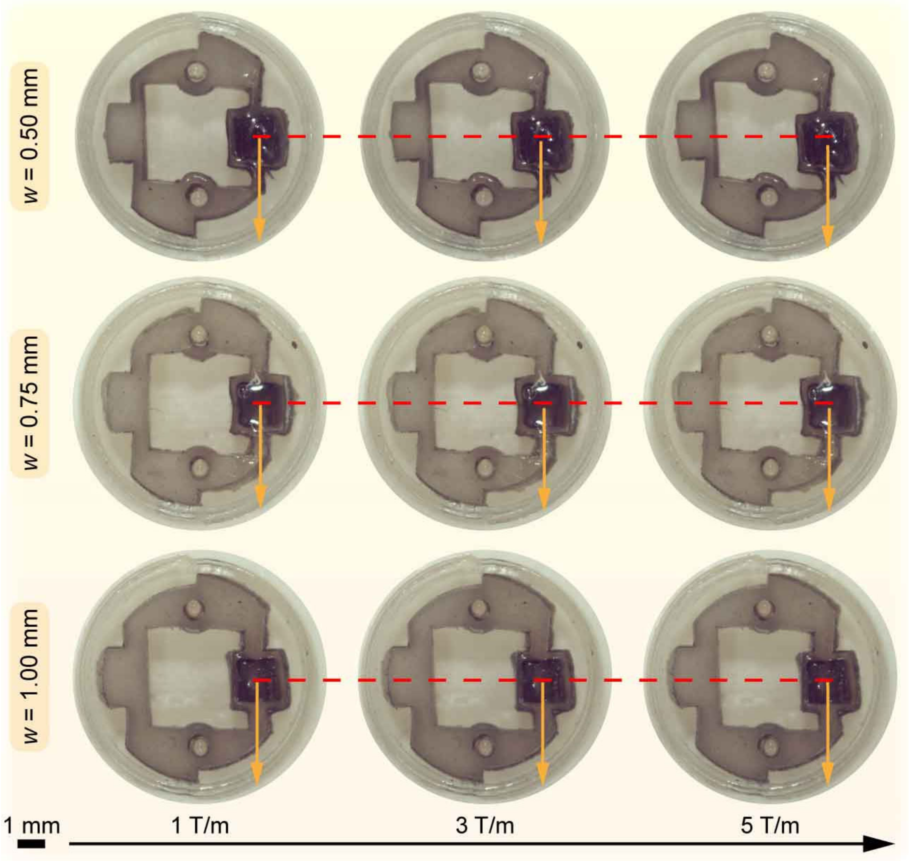

Fig. S3. Snapshots of the magnetic valves with different widths (w) under various magnetic gradients. $l = 0.75$ mm. It shows that the stiffness of the magnetic valve along the opening direction increases with the width value. The orange arrows indicate the direction of the magnetic force. Red dashed lines mark the reference positions.

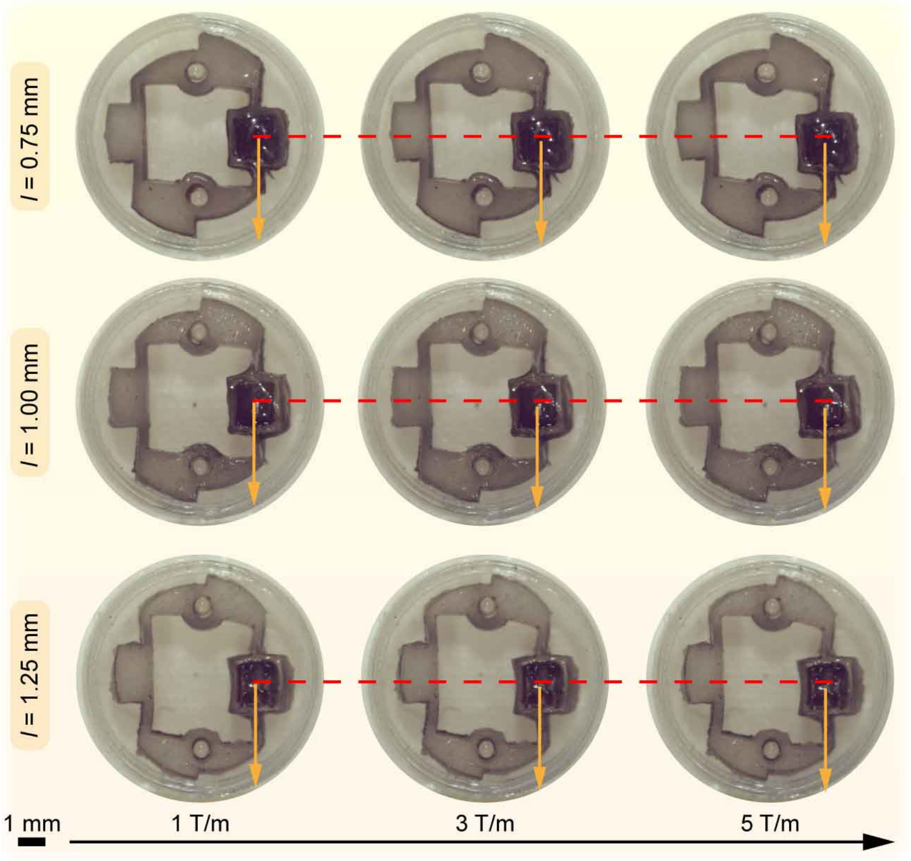

Fig. S4. Snapshots of the magnetic valves with different lengths (l) under various magnetic gradients. $w = 0.50$ mm. It shows that the stiffness of the magnetic valve along the opening direction decreases with increasing length. The orange arrows indicate the direction of the magnetic force. Red dashed lines mark the reference positions.

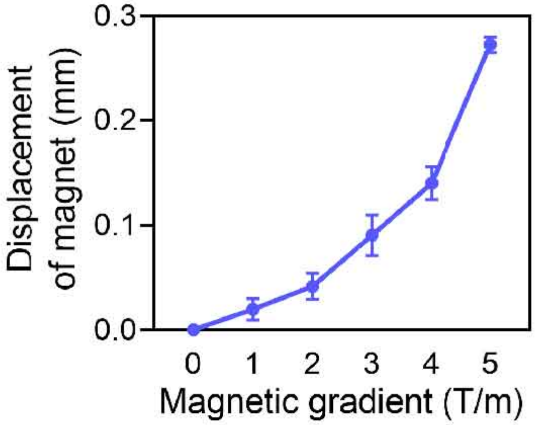

Fig. S5. Relationship between the deformation of the magnetic valve and the magnitude of the magnetic gradient. $w = 0.50$ mm and $l = 0.75$ mm. It shows that the deformation of the magnetic valve increases with the magnitude of the gradient magnetic field.

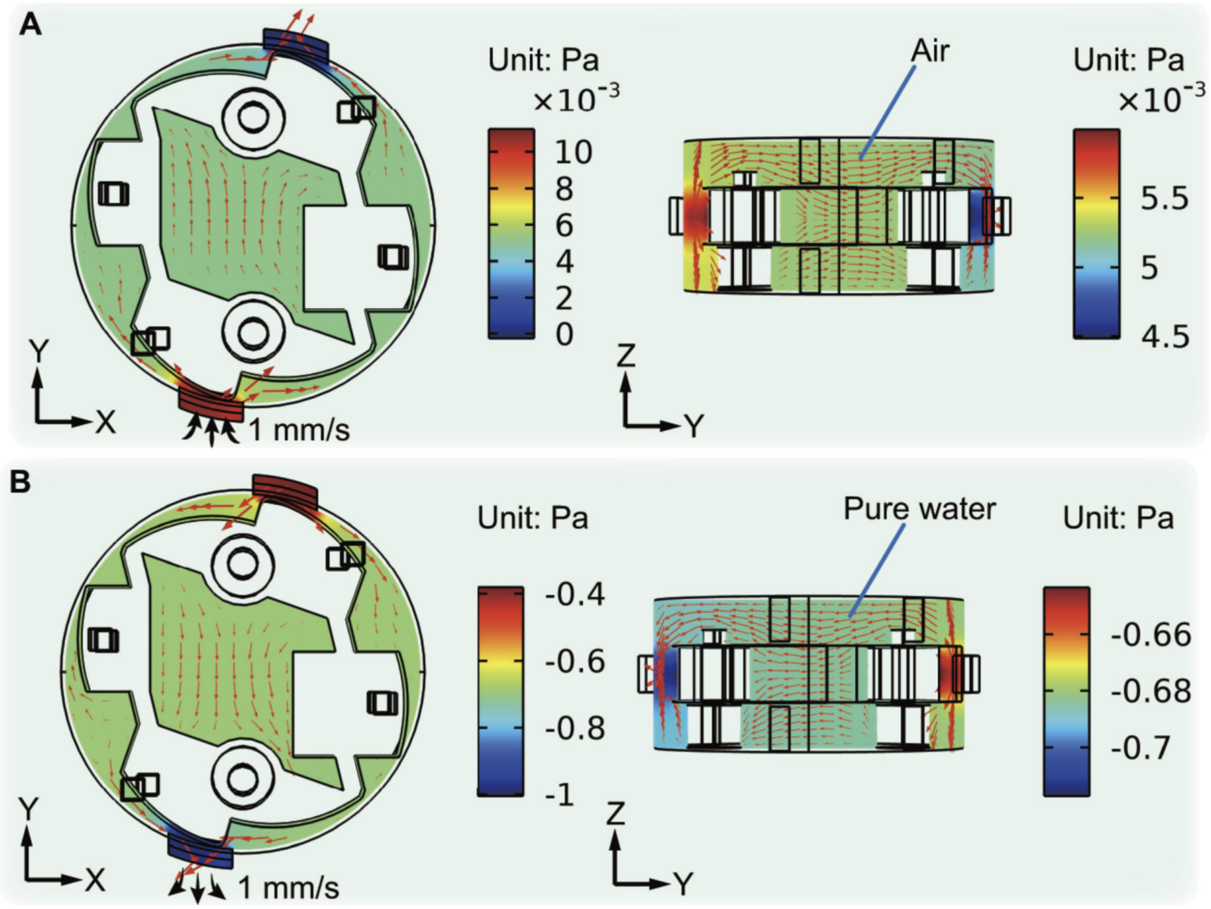

Fig. S6. Simulation results of the macabot. (A) Pressure distributions when sampling liquid. (B) Pressure distributions when the liquid was released. They are induced by the pressure difference between the upper and bottom vents. Red arrows indicate the liquid/air flow direction.

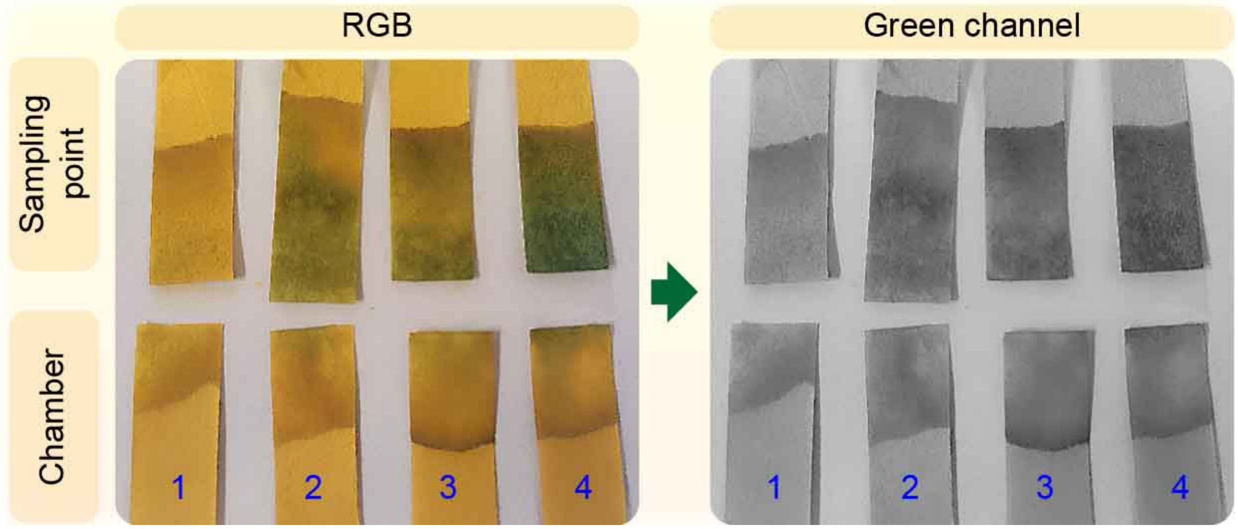

Fig. S7. Results of pH test papers for the liquids in the chambers and corresponding sampling points. It shows the pH values of the liquids in the chambers and the corresponding sampling points.

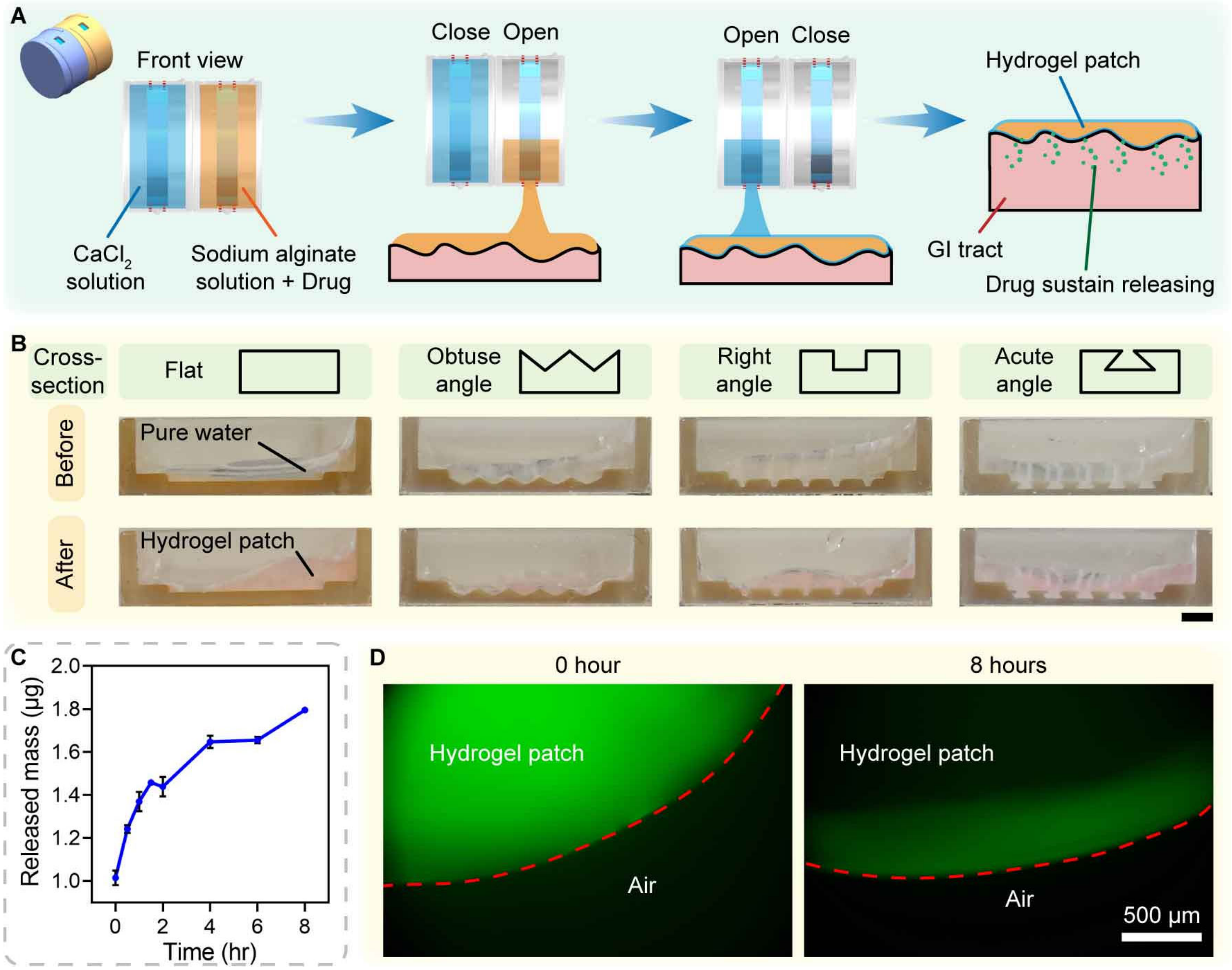

Fig. S8. Test results of the macabot for in situ forming hydrogel patch. (A) Schematic of the dual-chamber macabot system to generate a hydrogel patch in situ for sustained drug release. It is realized by releasing a sodium alginate solution (containing drug) and $CaCl_2$ solution in sequence. The drug-loaded hydrogel patch enables localized treatment of GI diseases at the lesion site. (B) Illustration of in situ forming results of the hydrogel patch on the interfaces with various shapes. Scale bar, 2 mm. (C) Sustained release test result of the generated hydrogel patch, showing that the released drug mass increases over time. (D) Fluorescence images of the hydrogel patch in Petri dish demonstrating the hydrogel patch's effectiveness for drug diffusion. The red curve indicates the boundary of the hydrogel patch.

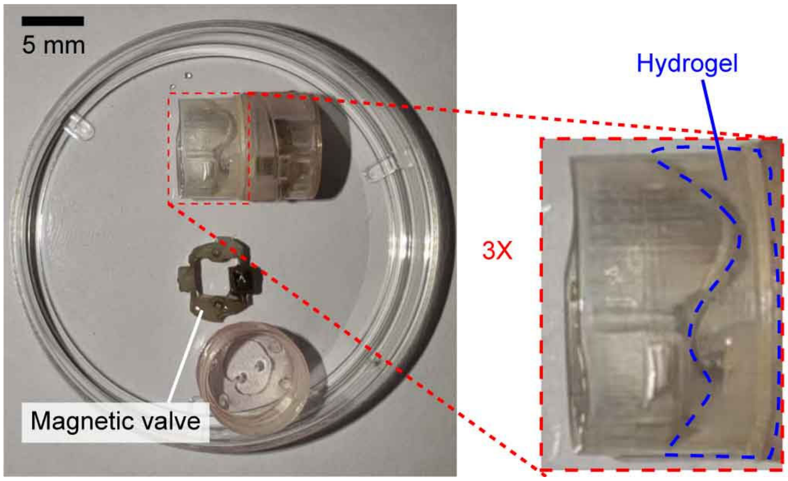

Fig. S9. Experimental result of releasing $CaCl_2$ solution and then the sodium alginate solution. It shows that the hydrogel is formed inside the chamber enclosed with the sodium alginate solution.

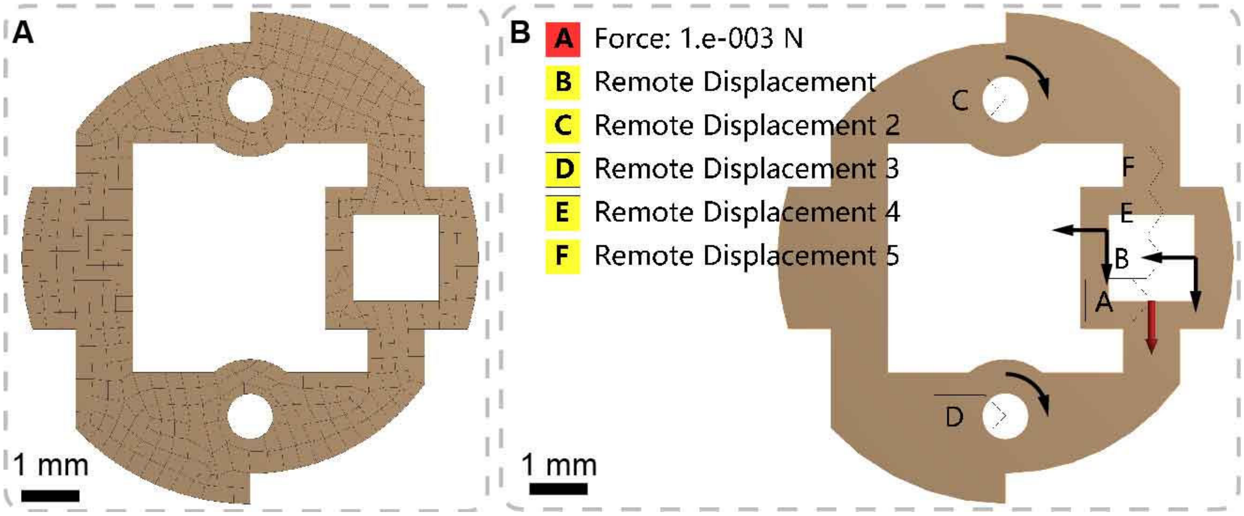

Fig. S10. Finite-element analysis simulation setup of the magnetic valve. (A) The mesh. (B) The setup of static structural analysis.

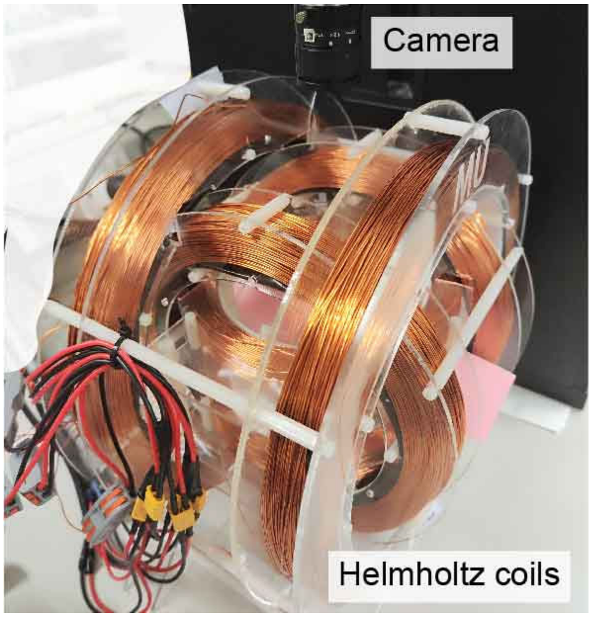

Fig. S11. Photograph of the custom-built 3D Helmholtz coils.

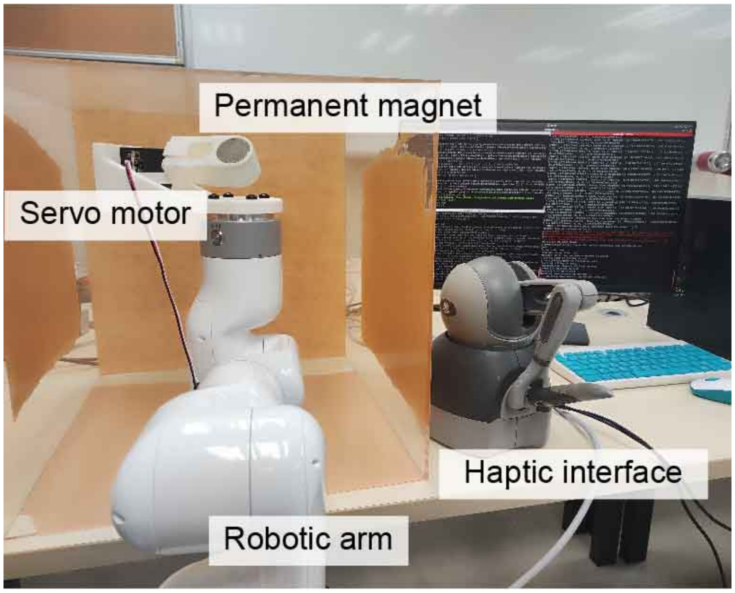

Fig. S12. Photograph of the developed teleoperated robotic system.

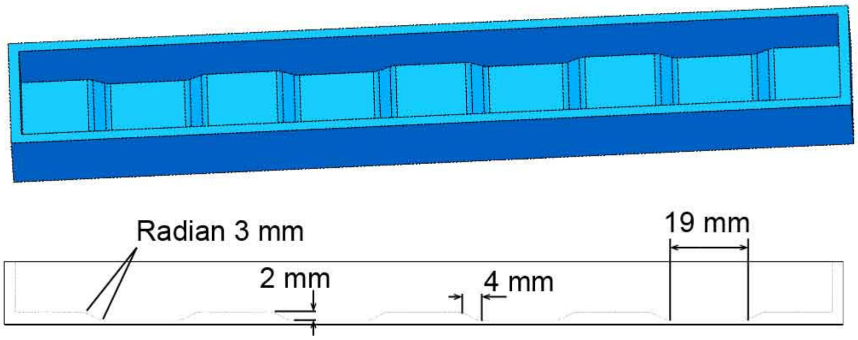

Fig. S13. Structural diagram of the passage with four pits.

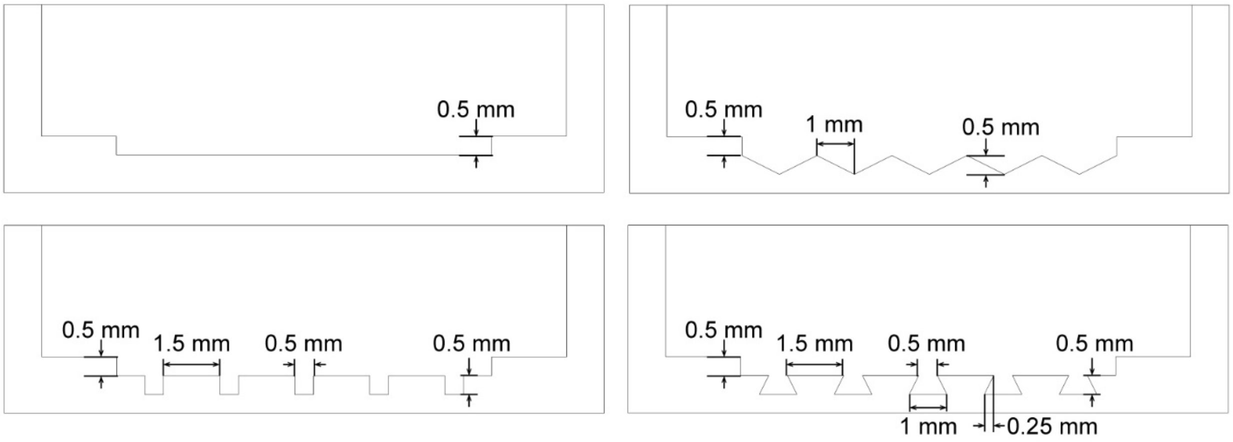

Fig. S14. Structural diagrams of four interfaces with various shapes.

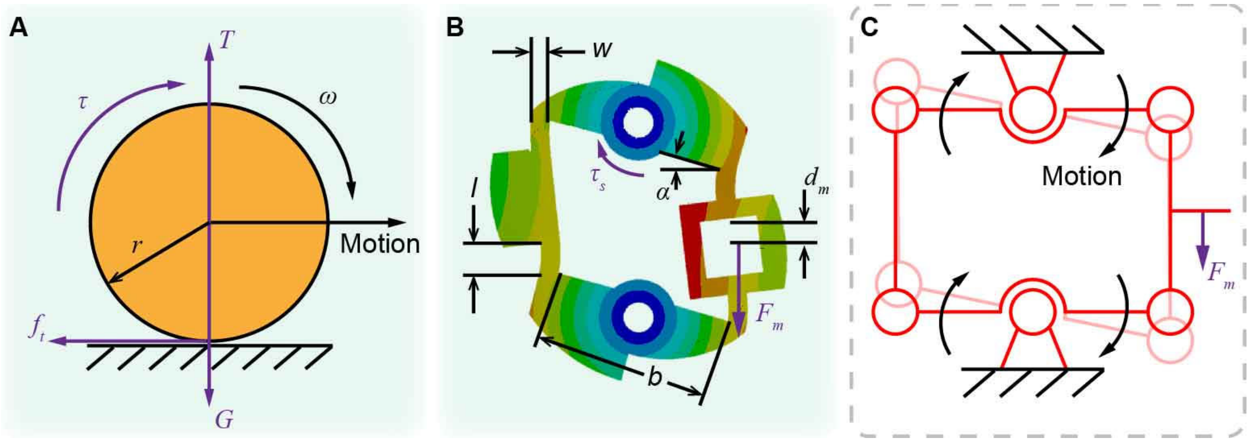

Fig. S15. Schematic diagram of mechanism design of a single-chamber capsule robot. (A) Rolling capsule robot. (B) Structural parameters of the magnetic valve. (C) Schematic diagram of the magnetic valve mechanism.

Supplementary Table

Table S1. Performance comparison of magnetic capsule robots in the literature.

Ref.	No. of chambers	Size (mm)	RDC	D/S/R	Actuation method	Triggering
(7)	1	Φ13.5 × 17	0.49	√/×/√	MT/-/MT	-
(28)	1	Φ12 × 33	0.26	√/× /√	MT/-/MT	-
(29)	1	Φ1.7	-	√/× /√	MT/-/MF	-
(30)	2	6.5 × 6.5 × 14	0.21	√/×/√	MF/-/MT	Separate
(31)	1	Φ15 × 30	0.15	√/× /√	MF/-/MT	-
(32)	1	Φ12 × 13	0.26	√/×/√	MT/-/MF	-
(33)	1	Φ13 × 20	-	√/×/√	MT/-/MF	-
(34)	1	Φ13	0.26	√/×/√	MT/-/MF	-
(35)	2	Φ14 × 28.9	0.34	√/×/√	MT/-/MT	Separate
(36)	4	Φ2.8 × 5<br>Φ0.84 × 1.5	0.32<br>0.08	√/×/√	MT/-/MT	Separate
(37)	2	Φ5.8 × 13<br>Φ8.4 × 19.5	0.35<br>0.42	√/√, L/√	MT/MT/MT	Sequence
(39)	1	Φ15 × 32	-	√/√, TI/×	MT/MF/-	-
(40)	1	Φ12× 32.2	-	√/√, TI/×	MT/MF/-	-
(41)	1	Φ18× 31.5	-	√/√, TI/×	MT/MF/-	-
(42)	1	Φ12× 31	-	√/√, TI/×	MF/MT/-	-
(43)	1	Φ14 × 20	0.10	×/√, L/×	-/MF/-	-
(44)	3	Φ11 × 26	0.02	√/√, L/×	MF/MT/-	Separate
(45)	6	Φ11 × 26	1.3×10^{-4}	√/√, L/×	MT/MF/-	Separate
(46)	1	Φ1.8 × 1.6	0.19	√/√, L/×	MT/MT/-	-
**Our work**	**4**	**Φ9.8 × 20.0**	**0.48**	√/√, L/√	**MT/MF/MF**	**Separate**

RDC: Ratio of inclusion volume to the total volume of the capsule.

D/S/R: Target delivery/sampling/releasing drug.

L/TI: Sampling liquid/tissue.

MT/MF: Magnetic torque/magnetic force.

Supplementary Movies

Movie S1. Locomotion test of the capsule robot.

Movie S2. Experimental study of sampling liquids in four pits.

Movie S3. Experimental study of releasing liquids.

Movie S4. *Ex-vivo* experimental study of the capsule robot.
